# Supplementary material for: Application of Pseudoinfectious Viruses in Transient Gene Expression in Mammalian Cells: Combining Efficient Expression with Regulatory Compliance
Source: Biomolecules. 2025 Feb 13;15(2):274. doi: 10.3390/biom15020274 (PMC11852456; doi:10.3390/biom15020274)
Supplement: Supplementary file 1 [file biomolecules-15-00274-s001.zip › biomolecules-3420148-supplementary.pdf]

Supplementary Materials to

Application of pseudoinfectious viruses in transient gene ex-pression: combining efficient expression with regulatory compliance

Gulzat Zauatbayeva [1], Tolganay Kulatay [1], Bakytkali Ingirbay [1], Zhanar Shakhmanova [1], Viktoriya Keyer [1], Mikhail Zaripov [2], Maral Zhumabekova [1], Alexandr V. Shustov *[1]

[1] National Center for Biotechnology, Kazakhstan; shustov@biocenter.kz

[2] Institute of Theoretical and Experimental Biophysics, Russia; mizaripov@mail.ru

***Correspondence**:

Alexandr V. Shustov, shustov@biocenter.kz , phone: +77024735305 (Watsup)

**G.Z.**: zauatbaeva@biocenter.kz	ORCID 0000-0003-1514-9302
**T.K.**: kulatay@biocenter.kz	ORCID 0009-0004-5885-8963
**B.I.**: ingirbay@biocenter.kz	ORCID 0000-0002-6915-8207
**Z.S.**: zhanar.shakhmanova@bk.ru	ORCID 0009-0000-0471-2388
**V.K.**: keer@biocenter.kz	ORCID 0000-0001-8885-2387
**M.Za.**: mizaripov@mail.ru	ORCID 0000-0001-5367-8868
**M.Zh.**: zhumabekova@biocenter.kz	0009-0002-9532-6232
**A.V.S.**: shustov@biocenter.kz	ORCID 0000-0001-9880-9382

```
                 VEEV subgenomic promoter                                                          GFP
1     TAACCTGAAT GGACTACGAC ATAGTCTAGT CCGCCAAGTC TAGAGCTTGC CGCCACCATG GTGAGCAAGG GCGAGGAGCT GTTCACCGGG GTGGTGCCCA
                                                           GFP
101   TCCTGGTCGA GCTGGACGGC GACGTAAACG GCCACAAGTT CAGCGTGTCC GGCGAGGGCG AGGGCGATGC CACCTACGGC AAGCTGACCC TGAAGTTCAT
                                                           GFP
201   CTGCACCACC GGCAAGCTGC CCGTGCCCTG GCCCACCCTC GTGACCACCC TGACCTACGG CGTGCAGTGC TTCAGCCGCT ACCCCGACCA CATGAAGCAG
                                                           GFP
301   CACGACTTCT TCAAGTCCGC CATGCCCGAA GGCTACGTCC AGGAGCGCAC CATCTTCTTC AAGGACGACG GCAACTACAA GACCCGCGCC GAGGTGAAGT
                                                           GFP
401   TCGAGGGCGA CACCCTGGTG AACCGCATCG AGCTGAAGGG CATCGACTTC AAGGAGGACG GCAACATCCT GGGGCACAAG CTGGAGTACA ACTACAACAG
                                                           GFP
501   CCACAACGTC TATATCATGG CCGACAAGCA GAAGAACGGC ATCAAGGTGA ACTTCAAGAT CCGCCACAAC ATCGAGGACG GCAGCGTGCA GCTCGCCGAC
                                                           GFP
601   CACTACCAGC AGAACACCCC CATCGGCGAC GGCCCCGTGC TGCTGCCCGA CAACCACTAC CTGAGCACCC AGTCCGCCCT GAGCAAAGAC CCCAACGAGA
                                          GFP                                                            FMDV 2A
701   AGCGCGATCA CATGGTCCTG CTGGAGTTCG TGACCGCCGC CGGGATCACT CTCGGCATGG ACGAGCTGTA CAAGCTTAAC TTCGACCTGC TGAAGCTCGC
                 FMDV 2A                                                         SEAP Signal peptide
801   CGGCGACGTG GAGAGCAACC CTGGCCCTTT CATGATTCTG GGCCCTGCA TGCTGCTGCT GCTGCTGCTG CTGGGCCTGA GGCTACAGCT CTCCCTGGGC
                                                  Secreted His-tagged SEAP
901   ATCATCCCAG TTGAGGAGGA GAACCCGGAC TTCTGGAACC GCGAGGCAGC CGAGGCCCTG GGTGCCGCCA AGAAGCTGCA GCCTGCACAG ACAGCCGCCA
                                                  Secreted His-tagged SEAP
1001  AGAACCTCAT CATCTTCCTG GGCGATGGGA TGGGGGTGTC TACGGTGACA GCTGCCAGGA TCCTAAAAGG GCAGAAGAAG GACAAACTGG GGCCTGAGAT
                                                  Secreted His-tagged SEAP
1101  ACCCCTGGCT ATGGACCGCT TCCCATATGT GGCTCTGTCC AAGACATACA ATGTAGACAA ACATGTGCCA GACAGTGGAG CCACAGCCAC GGCCTACCTG
                                                  Secreted His-tagged SEAP
1201  TGCGGGGTCA AGGGCAACTT CCAGACCATT GGCTTGAGTG CAGCCGCCCG CTTTAACCAG TGCAACACGA CACGCGGCAA CGAGGTCATC TCCGTGATGA
                                                  Secreted His-tagged SEAP
1301  ATCGGGCCAA GAAAGCAGGG AAGTCAGTGG GAGTGGTAAC CACCACACGA GTGCAGCACG CCTCGCCAGC CGGCACCTAC GCCCACACGG TGAACCGCAA
                                                  Secreted His-tagged SEAP
1401  CTGGTACTCG GACGCCGACG TGCCTGCCTC GGCCCGCCAG GAGGGGTGCC AGGACATCGC TACGCAGCTC ATCTCCAACA TGGACATTGA TGTGATCCTG
                                                  Secreted His-tagged SEAP
1501  GGTGGAGGCC GAAAGTACAT GTTTCGCATG GGAACCCCAG ACCCTGAGTA CCCAGATGAC TACAGCCAAG GTGGGACCAG GCTGGACGGG AAGAATCTGG
                                                  Secreted His-tagged SEAP
1601  TGCAGGAATG GCTGGCGAAG CGCCAGGGTG CCCGGTATGT GTGGAACCGC ACTGAGCTCA TGCAGGCTTC CCTGGACCCG TCTGTGACCC ATCTCATGGG
                                                  Secreted His-tagged SEAP
1701  TCTCTTTGAG CCTGGAGACA TGAAATACGA GATCCACCGA GACTCCACAC TGGACCCCTC CCTGATGGAG ATGACAGAGG CTGCCCTGCG CCTGCTGAGC
                                                  Secreted His-tagged SEAP
1801  AGGAACCCCC GCGGCTTCTT CCTCTTCGTG GAGGGTGGTC GCATCGACCA CGGTCATCAC GAAAGCAGGG CTTACCGGGC ACTGACTGAG ACGATCATGT
                                                  Secreted His-tagged SEAP
1901  TCGACGACGC CATTGAGAGG GCGGGCCAGC TCACCAGCGA GGAGGACACG CTGAGCCTCG TCACTGCCGA CCACTCCCAC GTCTTCTCCT TCGGAGGCTA
                                                  Secreted His-tagged SEAP
2001  CCCCCTGCGA GGGAGCTCCA TCTTCGGGCT GGCCCCTGGC AAGGCCCGGG ACAGGAAGGC CTACACGGTC CTCCTATACG GAAACGGTCC AGGCTATGTG
                                                  Secreted His-tagged SEAP
2101  CTCAAGGACG GCGCCCGGCC GGATGTTACC GAGAGCGAGA GCGGGAGCCC CGAGTATCGG CAGCAGTCAG CAGTGCCCCT GGACGAAGAG ACCCACGCAG
                                                  Secreted His-tagged SEAP
2201  GCGAGGACGT GGCGGTGTTC GCGCGCGGCC CGCAGGCGCA CCTGGTTCAC GGCGTGCAGG AGCAGACCTT CATAGCGCAC GTCATGGCCT TCGCCGCCTG
                                                  Secreted His-tagged SEAP
2301  CCTGGAGCCC TACACCGCCT GCGACCTGGC GCCCCCCGCC GGCACCACCG ACGCCGCGCA CCCGGGGCGG TCCCGGTCCA AGCGTCTGGA TGGGGCCATG
                                 Secreted His-tagged SEAP
                 10xHis-tag
2401  GGTCATCATC ATCACCATCA CCACCACCAT CACTCGTCCG GTCACATTGA GGGGCGCCAT ATGTAA
```

Figure S1. Mapped sequence of the gene cassette GFP-2A-SEAP. Component genes are labeled above the sequence.

```
                                        GFP
  1   MVSKGEELFT GVVPILVELD GDVNGHKFSV SGEGEGDATY GKLTLKFICT
                                        GFP
 51   TGKLPVPWPT LVTTLTYGVQ CFSRYPDHMK QHDFFKSAMP EGYVQERTIF
                                        GFP
101   FKDDGNYKTR AEVKFEGDTL VNRIELKGID FKEDGNILGH KLEYNYNSHN
                                        GFP
151   VYIMADKQKN GIKVNFKIRH NIEDGSVQLA DHYQQNTPIG DGPVLLPDNH
                                  GFP                      FMDV 2A
201   YLSTQSALSK DPNEKRDHMV LLEFVTAAGI TLGMDELYKL NFDLLKLAGD
                                                 Secreted His-tagged SEAP
      FMDV 2A              Signal peptide
251   VESNPGPFMI LGPCMLLLLL LLGLRLQLSL GIIPVEEENP DFWNREAAEA
                                Secreted His-tagged SEAP
301   LGAAKKLQPA QTAAKNLIIF LGDGMGVSTV TAARILKGQK KDKLGPEIPL
                                Secreted His-tagged SEAP
351   AMDRFPYVAL SKTYNVDKHV PDSGATATAY LCGVKGNFQT IGLSAAARFN
                                Secreted His-tagged SEAP
401   QCNTTRGNEV ISVMNRAKKA GKSVGVVTTT RVQHASPAGT YAHTVNRNWY
                                Secreted His-tagged SEAP
451   SDADVPASAR QEGCQDIATQ LISNMDIDVI LGGGRKYMFR MGTPDPEYPD
                                Secreted His-tagged SEAP
501   DYSQGGTRLD GKNLVQEWLA KRQGARYVWN RTELMQASLD PSVTHLMGLF
                                Secreted His-tagged SEAP
551   EPGDMKYEIH RDSTLDPSLM EMTEAALRLL SRNPRGFFLF VEGGRIDHGH
                                Secreted His-tagged SEAP
601   HESRAYRALT ETIMFDDAIE RAGQLTSEED TLSLVTADHS HVFSFGGYPL
                                Secreted His-tagged SEAP
651   RGSSIFGLAP GKARDRKAYT VLLYGNGPGY VLKDGARPDV TESESGSPEY
                                Secreted His-tagged SEAP
701   RQQSAVPLDE ETHAGEDVAV FARGPQAHLV HGVQEQTFIA HVMAFAACLE
                                                   10xHis tag
                                Secreted His-tagged SEAP
751   PYTACDLAPP AGTTDAAHPG RSRSKRLDGA MGHHHHHHHH HHSSGHIEGR
      Sec
801   HM
```

Figure S2. Mapped amino acid sequence of the fusion protein encoded in cassette GFP-2A-SEAP. Individual proteins are labeled above the sequence.

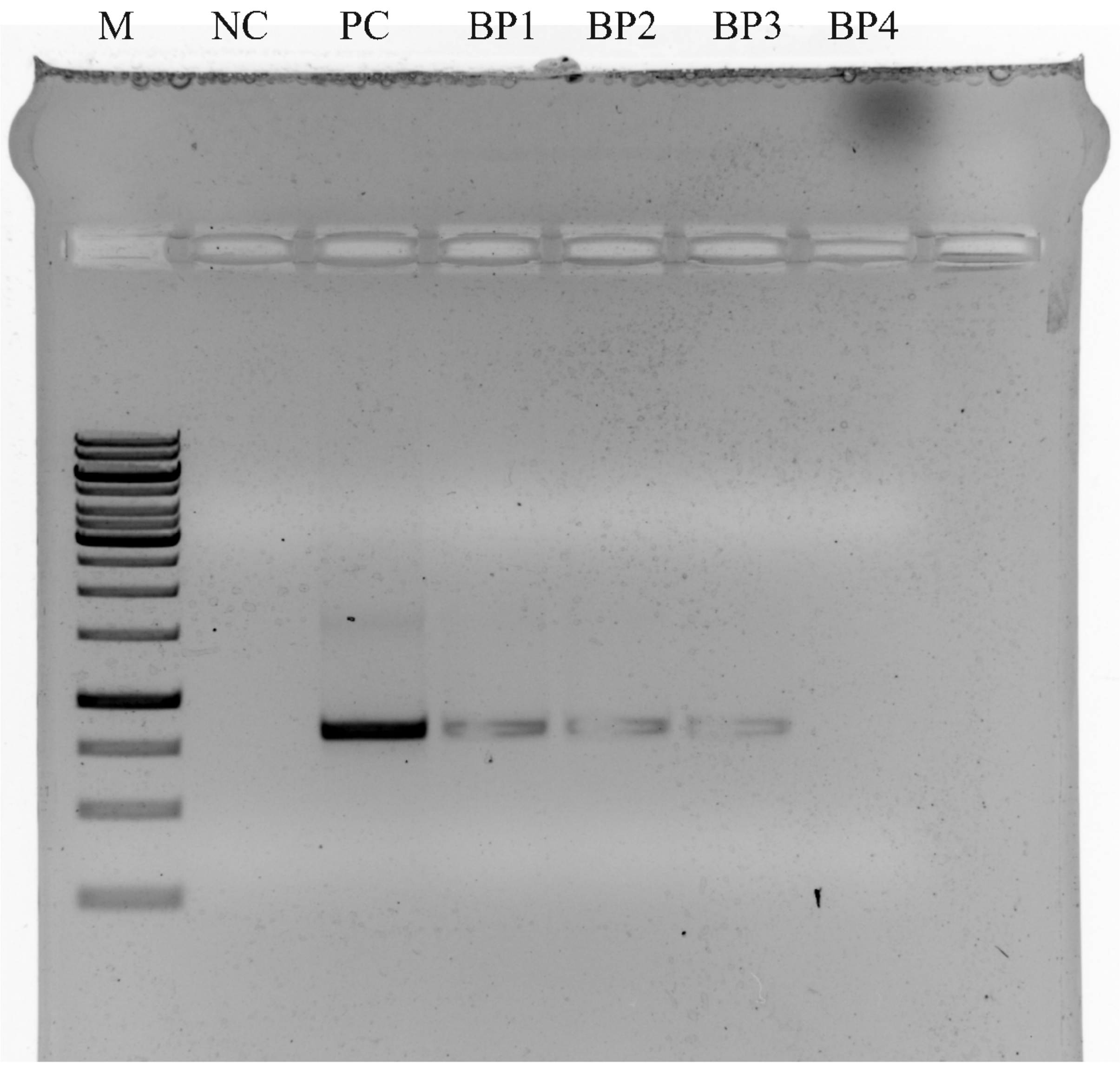

Figure S3. Experimental results for the assessment of replication-competent virus in a PIV particles preparation. Four blind passages were performed in HEK293FT cells, starting with a PIV particles preparation as the initial sample, as described in the Materials and Methods. Media collected from infected cells were analyzed by RT-PCR to amplify a fragment of the viral nsP2 gene, an essential component of the viral replicase. Lanes: M, DNA marker O'GeneRuler 100–10,000 (ThermoScientific, Cat. SM0331); NC, Negative control (medium from uninfected cells); PC, Positive control (PIV particle sample); BP1-4, amplification products from material present in the conditioned medium collected from infected cells at passages 1-4. The expected size of the amplification product is 839 bp. Vector particles were detected in BP1-3 but not in BP4.

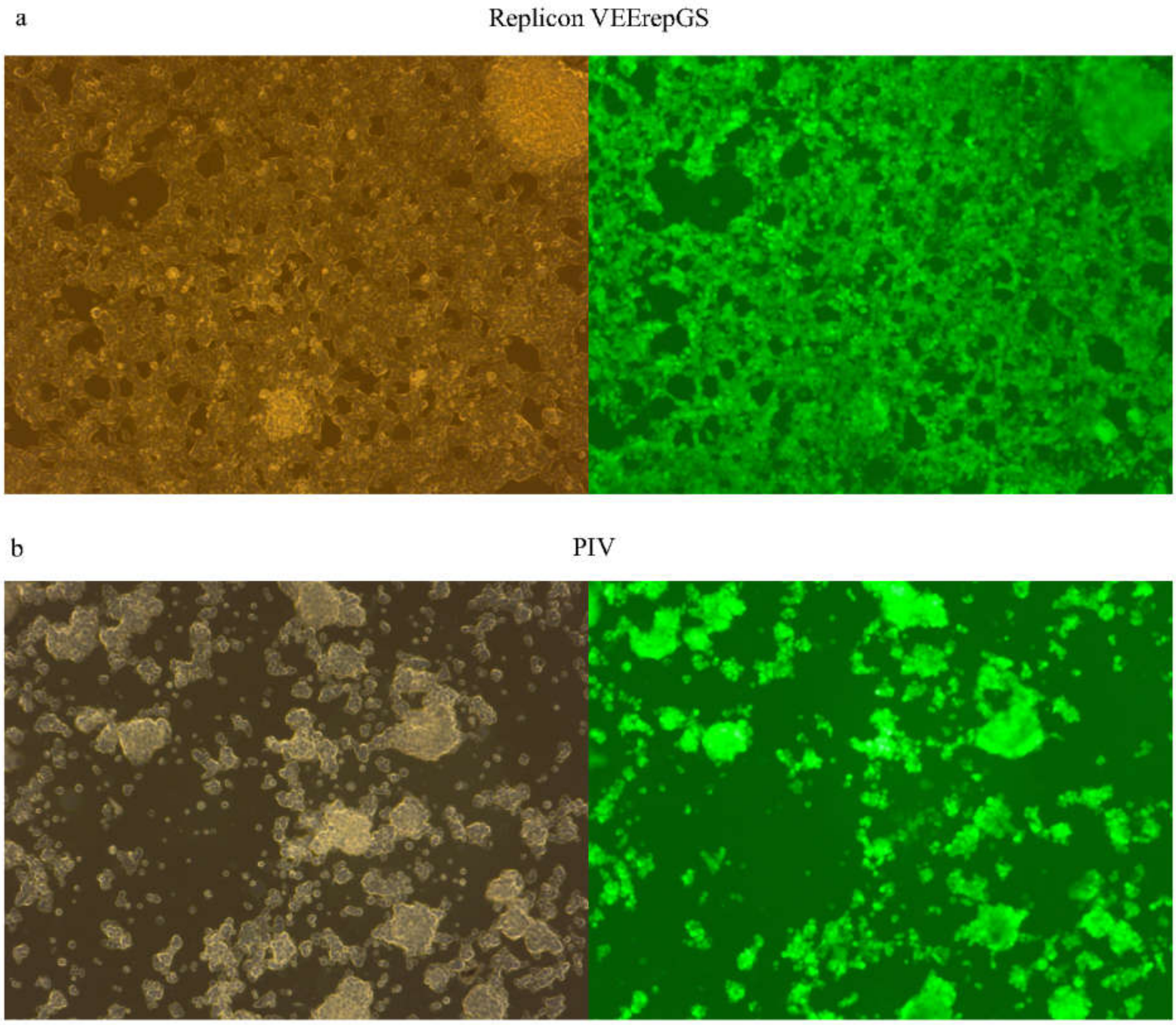

Figure S4. Photographs of cultures infected with the replicon (panel a) or PIV (panel b). The images were captured 48 hours post-infection. Cytopathic effects of PIV are evident, with cells becoming rounded and detaching from the bottom of the flask.

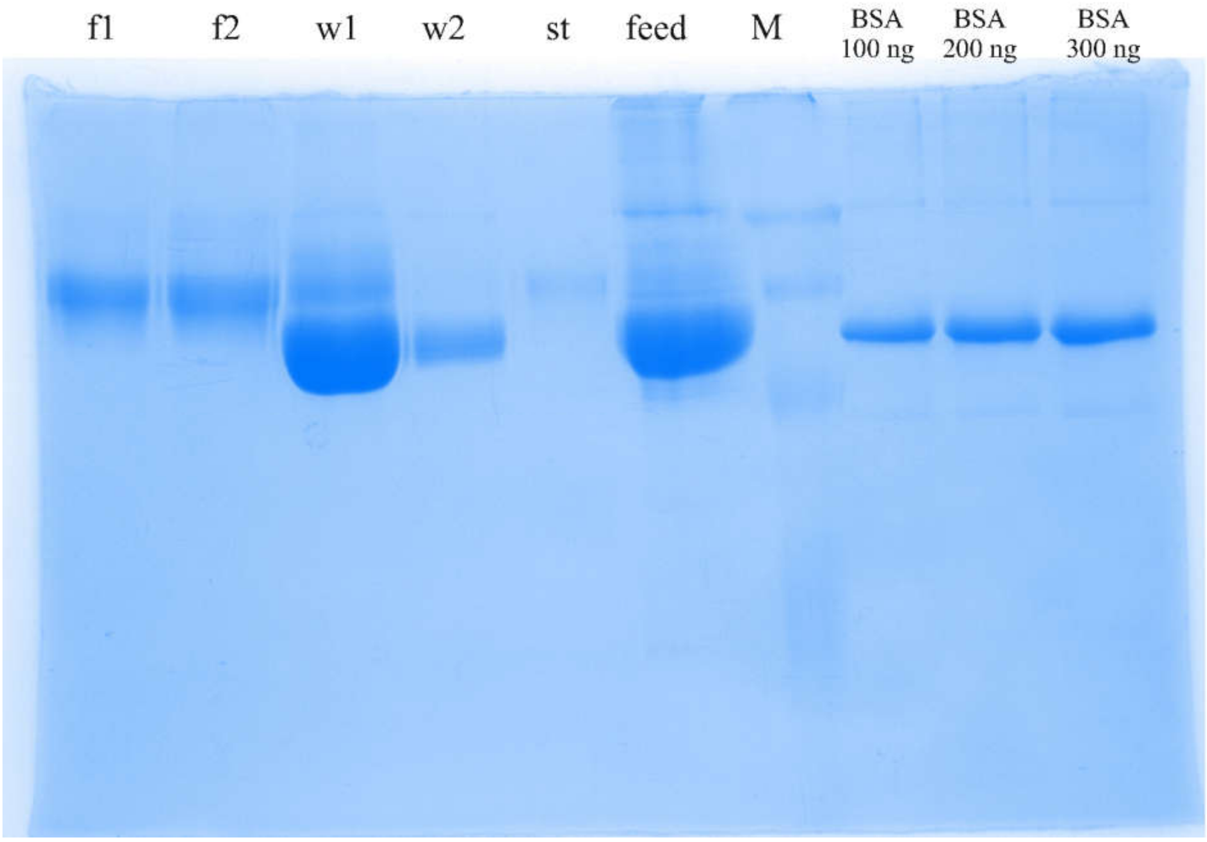

Figure S5. SDS-PAGE of samples collected during SEAP purification from culture medium. Lanes: f1, f2 represent fractions eluted with 250 mM imidazole; W1, W2 correspond to material washed from IMAC resin with two successive washes using 50 mM imidazole; St is the material obtained after stripping the resin with 50 mM EDTA; Feed is the spent medium before IMAC. M is the molecular mass marker. Bovine serum albumin (commercially pure preparation) was loaded as a concentration marker at 100 ng, 200 ng, and 300 ng. Further details are provided in the article.

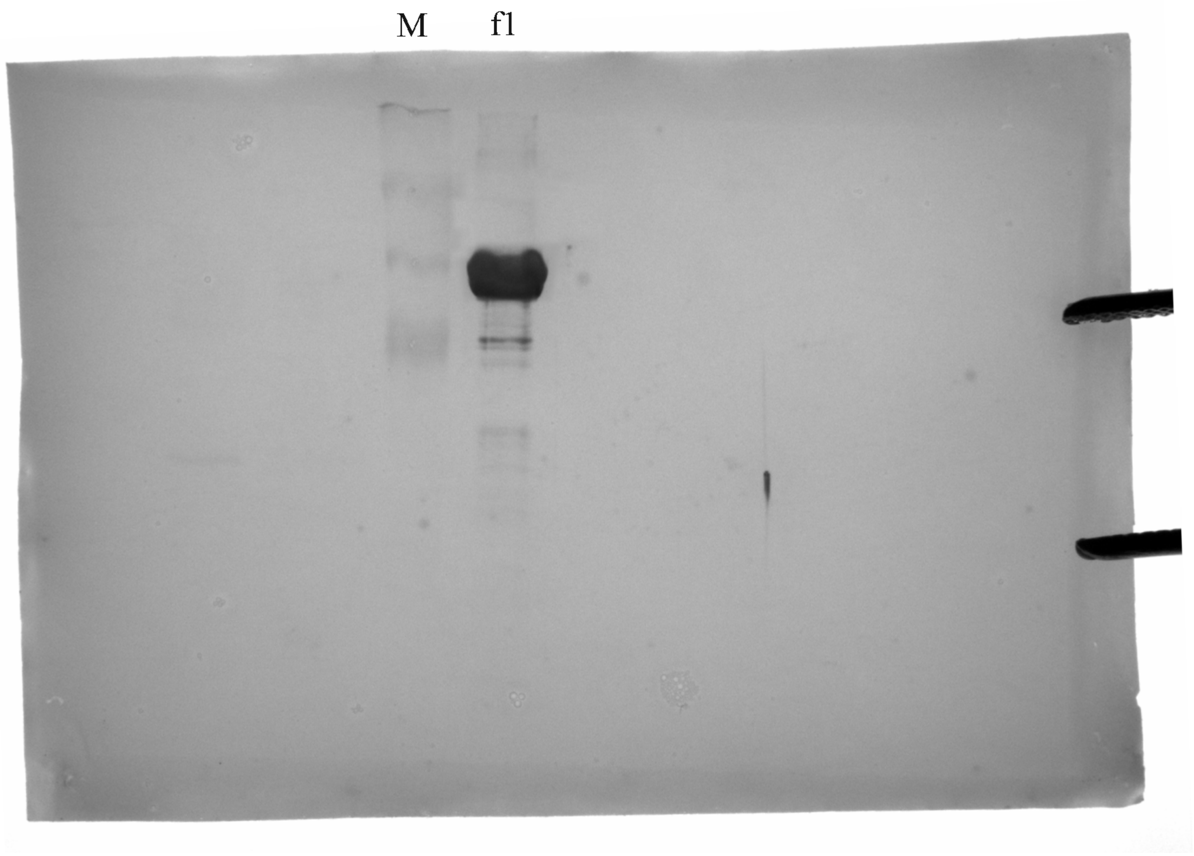

Figure S6. Western blot probed with anti-His-tag antibody. Lanes: f1 represents the first fraction eluted with 250 mM imidazole; M is the molecular mass marker, with bands visible on the membrane. Additional details are provided in the article.
